# High Resolution Melt analysis for mutation screening in *PKD1 *and *PKD2 *

**DOI:** 10.1186/1471-2369-12-57

**Published:** 2011-10-18

**Authors:** Stanislas Bataille, Yvon Berland, Michel Fontes, Stéphane Burtey

**Affiliations:** 1 EA 4263 Thérapie des maladies génétiques, Faculté de médecine, Université de la méditerranée, Boulevard Jean Moulin 13005 Marseille, France; 2 Centre de néphrologie et transplantation rénale, Hôpital de la Conception, 147 Boulevard Baille 13005 Marseille, France

## Abstract

**Background:**

Autosomal dominant polycystic kidney disease (ADPKD) is the most common hereditary kidney disorder. It is characterized by focal development and progressive enlargement of renal cysts leading to end-stage renal disease. *PKD1 *and *PKD2 *have been implicated in ADPKD pathogenesis but genetic features and the size of *PKD1 *make genetic diagnosis tedious.

**Methods:**

We aim to prove that high resolution melt analysis (HRM), a recent technique in molecular biology, can facilitate molecular diagnosis of ADPKD. We screened for mutations in *PKD1 *and *PKD2 *with HRM in 37 unrelated patients with ADPKD.

**Results:**

We identified 440 sequence variants in the 37 patients. One hundred and thirty eight were different. We found 28 pathogenic mutations (25 in *PKD1 *and 3 in *PKD2 *) within 28 different patients, which is a diagnosis rate of 75% consistent with literature mean direct sequencing diagnosis rate. We describe 52 new sequence variants in *PKD1 *and two in *PKD2*.

**Conclusion:**

HRM analysis is a sensitive and specific method for molecular diagnosis of ADPKD. HRM analysis is also costless and time sparing. Thus, this method is efficient and might be used for mutation pre-screening in ADPKD genes.

## Background

Autosomal dominant polycystic kidney disease (ADPKD) is a hereditary kidney disorder affecting approximately one in 500 to one in 1000 human live births [1]. It is characterized by focal development and progressive enlargement of renal cysts, leading to end-stage renal disease (ESRD) [2].

ADPKD is genetically heterogeneous and involves two genes, *PKD1 *(MIM 601313, chromosome region 16p13.3) [3] and *PKD2 *(MIM 173910, 4q21-22) [4]. Mutations in *PKD1 *account for approximately 85% of ADPKD cases and are associated with a more severe disease than *PKD2 *. The median age at onset of ESRD is 54.3 [52.7-55.9] years for individuals with mutation in *PKD1 *compared to 74.0 [67.2-80.8] years in *PKD2 *[5]. It has been hypothesized that a third gene could be implicated in ADPKD [6] but there is no evidence for this.

Genetic analysis of ADPKD is difficult owing to the existence of at least two distinct genes that can cause disease and the lack of an exhaustive list of *PKD1 *and *PKD2 *mutations that are associated with it. Genomic features of *PKD1 *also cause difficulty in identifying sequence variants [7]. The open reading frame of *PKD1 *is approximately 13 kb split in 46 exons. Exons 1-33 are duplicated six times at the *HG *locus on a proximal position on chromosome 16p. The homology between the 5' region of *PKD1 *and these six pseudo genes ranges from 94.5 to 96.7% in the duplicated area and almost 100% for the coding sequence [8]. Locus-specific amplifications of *PKD1 *are required for analysis which complicates genetic diagnosis [9]. Furthermore, *PKD1 *is a highly polymorphic gene with numerous sequence variants [10,11] (Human Gene Mutation Database [HGMD]; http://www.hgmd.cf.ac.uk/ac/index.php[12]). Single nucleotide variants in the coding regions of the PKD genes could result in the development of ADPKD. Therefore, a strategy has been proposed to investigate the pathogenic significance of sequence variants in *PKD1 *[11].

Definitive diagnosis of ADPKD is based on an age-specific cystic renal phenotype and a positive family history [13]. Diagnosis of ADPKD in younger patients can be difficult as renal ultrasonography can be inconclusive and if the family history is unknown. Molecular diagnosis could be of use in providing a definitive diagnosis.

High Resolution Melt (HRM) analysis, a recent advance in molecular biology used to detect variants in DNA sequences [14], has replaced dHPLC, the reference screening method, for analyzing genetic variants of *BRCA1 *and *BRCA2 *[15]. There are major drawbacks with dHPLC including chemical waste, high maintenance costs and the need for post-PCR manipulations. Moreover, dHPLC does not allow high-throughput sequence variation screening.

HRM is based on the analysis of the melting curve of a real-time PCR amplicon. Monitoring the melting curve of the amplicon involves detecting the change in fluorescence intensity induced by the release of an intercalating DNA dye from a DNA duplex as it denatures at high temperatures. Unlike dHPLC, this technique allows high throughput screening. Even if the sequence is the endpoint of molecular analysis, a pre-screening step always reduces the cost compared to the whole gene sequencing [16]. Therefore, with regard to *PKD1 *and *PKD2 *, HRM could be a rapid and cost effective technology for routine genetic analysis.

We show the results of an HRM screening strategy to identify sequence variants within *PKD1 *and *PKD2 *in 37 ADPKD patients and demonstrate that HRM is accurate to study ADPKD genes.

## Methods

### DNA samples

Thirty-seven unrelated patients from the Nephrology center of Marseille were screened for sequence variants in *PKD1 *and *PKD2 *. They all fulfilled the ADPKD unified diagnosis criteria [17]. The cohort is composed of 14 females and 23 males. The mean age is 51 years+/-11. They had normal renal function (2 patients) or chronic renal failure (6 patients) or end-stage renal disease (29 patients). Fifty healthy individuals are the control group. They are from the same area and ethnic group as patients. Genomic DNA was extracted from lymphocytes harvested by venous blood puncture after written informed consent. Quantification and quality of all experimental DNA samples were assessed using Nanodrop^® ^Technology (Coleman Technologies, Orlando, FL). DNA working solutions were diluted in ultrapure water to obtain the correct dilution. The study was conducted in compliance with the Helsinki Declaration and was approved by the Comité de Protection des Personnes (CPP) Sud-Méditerranée 2.

### Long Range PCR

*PKD1 *screening previously required amplification of five specific long range (LR) PCR with primers designed in the *PKD1 *sequence differing from the homologues; this step ensures to screen for sequence variants in *PKD1 *, not in homologues. Long range PCR assays were performed as described previously [18], LR1 amplified exon 1, LR2 amplified exon 2 to 12, LR3 amplified exon 13 to 15D, LR4 amplified exon 15E to 22, LR5 amplified exon 22 to 32. A sixth 7,838 bp LR PCR was designed to make HRM analysis for *PKD1 *exons 36 to 46 more specific with the forward and reverse primers: 5'-ACCTTCCCTCTAGGGAGGGAGCA-3' and 3'-GGCCAAGCTCGCATCCAAGCA-5'. Amplification was performed using the GeneAmp High Fidelity PCR System (Applied Biosystems, Foster City, CA) in a final volume of 50 µl containing 100 ng gDNA, 2.5U enzyme, 15 µM of each primer, 5 mM dNTP, 5 µl manufacturer's supplied buffer, 2 µl supplied MgCl_2 _and 10% DMSO. Cycling conditions were 95°C for 3 min, followed by 34 cycles at 96°C for 30 s, 62°C for 30 s, 68°C for 8 min, and a last step of 7 min at 72°C. All LR PCR products were verified on ethidium bromide stained gels.

### Primers

We used oligonucleotide primers described in the literature to perform PCR on each *PKD1 *and *PKD2 *exons and on exon-intron junctions, which is important in order to identify variants affecting mRNA splicing. Primers from Tan et al. [18] and from Rossetti et al. [10] were used for *PKD1 *analysis; primers from Tan et al. [18] and from Hayashi et al. [19] were used for *PKD2 *. Forty-three out of 46 exons of *PKD1 *were screened in 56 amplicons; 14 out of 15 exons of *PKD2 *were screened in 14 amplicons. Exons 1, 42, 43 of *PKD1 *and exon 1 of *PKD2 *could not be screened by HRM and were sequenced. Details of all primers are available in additional file 1.

### Real Time PCR and HRM conditions

Amplification and HRM were performed in 20 µl volumes in a LightCycler 480 (Roche Applied Systems). The amplification mixture included 20 ng of genomic DNA or 5 µl of 1/500 diluted PCR long range amplification product as template, 1 µl of LightCycler 480 ResoLight Dye (Roche, Indianapolis, IN), 10 µL of LightCycler 480 Probes Master (Roche, Indianapolis, IN) and 5 µM of each primer.

Each run included an activation step at 95°C for 10 min followed by 45 amplification cycles of 15 s denaturation, 15 s annealing and 15 s, elongation at 72°C. Denaturation and annealing temperatures differed according to each fragment these are detailed in additional file 1.

First, the products were heated to 96°C for 1 min and temperature sample was reduced at 40°C for 1 min. HRM was carried out with the temperature rising at a rate of 1°C per second with 25 acquisitions per degree. Initial and final temperatures also differed among amplicons (additional file 1). All reactions were performed in 96-well microtiter plates. Each fragment was tested in 10 patients per run.

### HRM analysis

For HRM analysis, the melting curves were normalized and the temperature shifted (temp-shifted) so that samples were comparable. Modified curves were obtained with LC480 software in the gene-scanning module (version 1.3; Roche). The normalized and temp-shifted melting curves corresponded to the final curve after normalization. When an amplicon harbored a sequence variation, the normalized and temp-shifted melting curve had a different shape from wild-type amplicons.

In this study, normalization was manually adjusted; in a routine framework, normalization ranges are automatically filled out. Default software sensitivity setting was 30% in order to avoid false negative amplicons. Therefore, sensitivity was set by default to 40% for all amplicons.

Comparing several wild-type amplicons demonstrated that the derived normalized and temp-shifted difference plot varies within a range owing to small random differences. The variance is partly caused by the specificity of the primers. Every amplicon that had a different derived normalized temp-shifted curve from wild-type was sequenced.

### Reverse transcription analysis

To confirm the signification of the suspected splice mutation c.7210-5C>G, we performed RT-PCR from RNA extracted of leucocytes as previously described [20], briefly lymphocytes were isolated using a Ficoll gradient and total RNA was extracted using the Rneasy Qiagen kit, according to the manufacturer's instructions (Qiagen SA). Total RNA was resuspended in Rnase free water. A total of 5 μg of total RNA was reverse transcribed using a RT-PCR kit (RT Life Technology GIBCO BRL), according to the manufacturer's instructions, and 3 μl of the RT products were amplified using a standard hot start PCR technique using the primers, for *PKD1 *specific PCR (6F 5'-AGCGCAACTACTTGGAGGCCC-3' and 6R 5'-ACCACAACGGAGTTGGCGG-3') and we diluted this RT-PCR product to 1/1000 and performed a nested PCR with the following primers (SAB26F2 5'-CAGGCCAATGTGACGGTGG-3' and SAB26B3 5'-CAGTCGCATGCCTGCACTGC-3').

### Sequencing

Sequencing was used to confirm and investigate variants. All sequencing analysis was subcontracted to Cogenics^® ^(Cogenics, Meylan, France) and performed by Sanger sequencing method. Capillary sequencing is performed using Big Dye* Terminator chemistry and sequences are delineated with Applied Biosystems 3730 × l platforms (http://www.beckmangenomics.com/documents/services/GS_Sanger_Sequencing.pdf). Samples were prepared by diluting 20 µl PCR product in 30 µl of ultrapure water. Primers were diluted to obtain a concentration of 2 mM as recommended by the subcontractor.

### Sequence variation analysis and classification

All sequences were compared to the following references sequences NCBI RefSeq *PKD1 *: NM_000296.2; and *PKD2 *: NM_000297.2. The standard nomenclature recommended by HGVS (http://www.hgvs.org/mutnomen) was used to number nucleotides and name mutations.

For previously described sequence variants, we reported the clinical significance assessed in The Polycystic Kidney Disease Mutation Database accessible at http://pkdb.mayo.edu[12]. Pathogenic potential of novel mutations was assessed as follows: - nonsense or frameshift variants leading to a STOP codon as well as intronic mutations altering mRNA sequence proved with RT-PCR were considered as definitely pathogenic mutations. - intronic and synonymous variants that did not alter predicted splicing (http://www.fruitfly.org/seq_tools/splice.html) were considered as polymorphisms, the others as indeterminate mutations. - substitutions were evaluated using the scoring method described by Rossetti et al [11] and with PolyPhen, a software for interspecies sequence variations examination (http://genetics.bwh.harvard.edu/pph) [21]. When the two scores assessed a pathogenic variant the mutation was considered as probably pathogenic, when they assessed a non pathogenic variant it was considered as polymorphism. If the two tests were discordant, the variation was reported as indeterminate.

## Results

**Figure 1:**
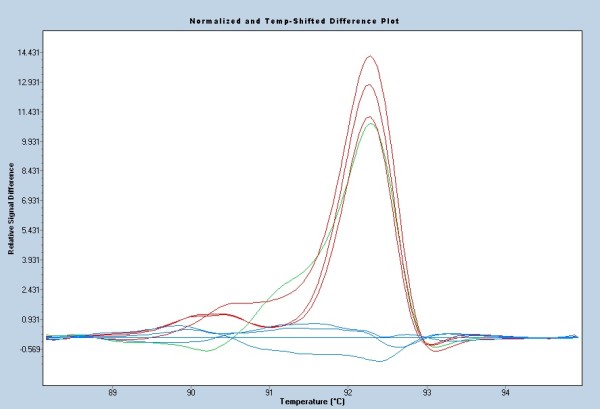
**Example of HRM analysis result: normalized and temp-shifted result curve of fragment 15D of 10 patients**. The three fragments with the red curves carry the same sequence variant p.Ala1555Ala (c.4665A>C), which is different from the fragment with the green curve p.Thr1558Thr (c.4674G>A). Fragments with the blue curve have no mutation.

Forty-three exons of *PKD1 *were analyzed using HRM in 56 fragments after PCR amplification. Of the 2,072 amplicons tested in the 37 patients, 302 (14.6%) were suspected to have a sequence variants compared to the wild-type amplicons analyzed (Figure 1). Each of the 302 amplicons was sequenced and 244 had at least one sequence variants. A total of 410 sequence variants were detected in *PKD1 *, corresponding to 11.1 ± 7.7 (mean ± SD) per patient; 3 exons were studied by direct sequencing. 129 different sequence variants were found.

Fourteen exons of *PKD2 *were analyzed in 14 fragments after PCR amplification. Of the 518 amplicons tested, HRM analysis identified 8 with sequence variants and 4 were shown to have at least one sequence variant after sequencing. A total of 30 sequence variants were detected in *PKD2*, one exon was studied by direct sequencing. 9 different sequence variants were found.

**Figure 2:**
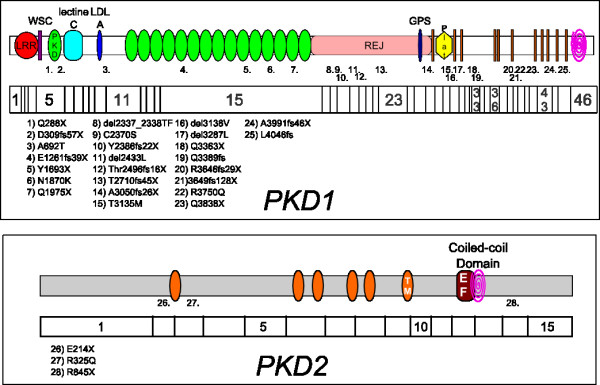
**Pathogenic mutations in *PKD1 *and *PKD2 ***. The pathogenic variants of *PKD1 *and *PKD2 *are positioned in a representation of genes and proteins.

Table 1 and Figure 2 show definitely pathogenic and probably pathogenic mutations. 25 mutations in *PKD1 *and 3 in *PKD2 *were identified in 28 patients. Of these 28 mutations, 9 were already described in literature. Of the 19 new mutations, 13 were identified as being definitely pathogenic because they lead to a STOP codon; 3 substitutions were scored as probably pathogenic (Additional file 2) as well as 1 in frame deletions, one intronic deletion and one intronic substitution.

**Table 1:** Mutations

DNA change	Protein change	Intron or exon	Fragment	Mutation type	Clinical significance	Reference
***PKD1 ***						

c.862C>T	p.Gln288X	Exon 5	5B	NONSENSE	Definitely pathogenic	Novel
c.926_939del14	p.Asp309fs	Exon 5	5B	FRAMESHIFT	Definitely pathogenic	Novel
c.2073G>A	p.Ala692Thr	Exon 10	10	SUBSTITUTION	Probably pathogenic	Novel
c.3783_3784insC	p.Glu1261fs	Exon 15	15B	FRAMESHIFT	Definitely pathogenic	Novel
c.5078C>A	p.Tyr1693X	Exon 15	15E	NONSENSE	Definitely pathogenic	Novel
c.5610C>G	p.Asn1870Lys	Exon 15	15F	SUBSTITUTION	Probably pathogenic	Novel
c.5923C>T	p.Gln1975X	Exon 15	15G	NONSENSE	Definitely pathogenic	Novel
c.7008_7013delCCTTCA	p.Thr2337_Phe2338del	Exon 16	16	DELETION	Probably pathogenic	Novel
c.7108T>A	p.Cys2370Ser	Exon 17	17	SUBSTITUTION	Probably pathogenic	[11]
c.7155_7156insT	p.Tyr2386fs	Exon 17	17	FRAMESHIFT	Definitely pathogenic	Novel
c.7298_7300delTGC	p.del2433Leu	Exon 18	18	DELETION	Probably pathogenic	[22]
c.7210-5C>G	pThr2496fs	Intron 18	19	SPLICE	Definitely pathogenic	**Novel**
c.8124_8127delGCCC	p.Thr2710fs	Exon 22	22	FRAMESHIFT	Definitely pathogenic	Novel
c.9147_9148insG	p.Ala3050fs	Exon 25	25	FRAMESHIFT	Definitely pathogenic	Novel
c.9403C>T	p.Thr3135Met	Exon 27	27	SUBSTITUTION	Probably pathogenic	Novel
c.9203_9205delGTG	p.del3138Val	Exon 27	27	DELETION	Probably pathogenic	[23]
c.9859_9861delCTC	p.del3287Leu	Exon 29	29	DELETION	Probably pathogenic	[34]
c.10086C>T	p.Gln3363X	Exon 31	31	NONSENSE	Definitely pathogenic	Novel
c.10167+24del19	p.Gln3389fs	Intron 31	31	SPLICE	Probably pathogenic	Novel
c.10932delC	p.Arg3646fs	Exon 37	37	FRAMESHIFT	Definitely pathogenic	Novel
c.10945_10952del8	p.3649fs	Exon 37	37	FRAMESHIFT	Definitely pathogenic	Novel
c.11249G>A	p.Arg3750Gln	Exon 39	39	SUBSTITUTION	Probably pathogenic	[32]
c.11512C>T	p.Gln3838X	Exon 41	41	NONSENSE	Definitely pathogenic	[35]
c.11972delC	p.Ala3991fs	Exon 43	43	FRAMESHIFT	Definitely pathogenic	Novel
c.12235+2T>C	p.Leu4046fs	Exon 44	44	SPLICE	Definitely pathogenic	[20]

			***PKD2 ***			

c.640G>T	p.Glu214X	Exon 2	2	NONSENSE	Definitely pathogenic	Novel
c.974G>A	p.Arg325Gln	Exon 4	4	SUBSTITUTION	Probably pathogenic	[11]
c.2533C>T	p.Arg845X	Exon 14	14	NONSENSE	Definitely pathogenic	[36]

No mutation was detected in more than one individual.

**Figure 3:**
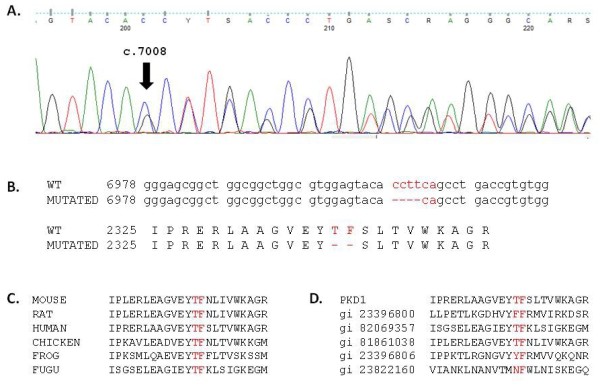
**Pathogenic variant p. Thr2337_Phe2338del**. A. Electrophoregram centered on sequence variant. The black arrow indicates the first deleted base. B. Alignments between wild type (WT) and mutated DNA sequences (top) and wild-type and mutated protein sequence (bottom). C. Alignments between deleted region and orthologs. D. Alignments between deleted region and conserved REJ domains.

The first in frame deletion, p.Thr2337_Phe2338del, was classified as probably pathogenic because the two residues are highly conserved during evolution, they are located in the functional REJ domain and this deletion was the only probably pathogenic mutation identified in this patient (Figure 3). The second deletion was p.Leu2433del. Since leucine 2433 is a conserved residue (in chicken, frog and takifugu but not in mouse or rat), this variant was also considered probably pathogenic. It was the only possible mutation causing disease identified in this patient. In addition, this mutation was previously described associated with lack of expression of PC-1 in primary cilia [22]. The two in frame deletions were not found in 100 control chromosomes tested.

**Figure 4:**
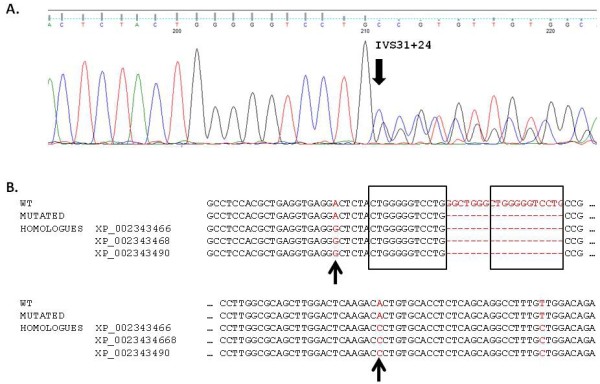
**Mutation. c.10167+24del19**. A. Electrophoregram centered on sequence variant. The black arrow indicates the location of the deletion. B. Alignments between wild type (WT), mutated and homologues DNA sequences. Black arrows show the flanking bases in the mutated sequence that is identical to WT but different from the homologues sequence. Black boxes represent a repeated 12 bp regions.

The 19 base pair intronic deletion in intron 31 (c.10167+24del19) was considered as probably pathogenic. Unfortunately, this patient was lost of follow up and no RT-PCR could be performed. A very similar deletion has previously been reported [23] as probably pathogenic in The Polycystic Kidney Disease Mutation Database. The deletion is present in three of the HG genes-*PKD1 *partial homologues on chromosome 16 (Figure 4) leading to a splice mutation associated with intron 31 retention. It was the only possible pathogenic mutation found in this patient and was not present in the 100 control chromosomes tested. The abnormal predicted splicing of intron 31 leads to a frameshift within exon 32. The presence of this deletion in three of the six *PKD1 *homologues is of interest as it is associated with retention of the intron. A possible hypothesis for the pathogenesis of this mutation is the occurrence of recombination between homologue and *PKD1*. If a crossover event has occurred, it must be a small crossover, because the deletion region is flanked by two bases differing from the homologues and identical to *PKD1 *(Figure 4). The mutation c.7210-5C>G was predicted to alter splicing *in silico *and no other indeterminate or pathogenic variation was found in this patient. We classified this mutation as pathogenic after we performed a specific RT-PCR as previously described [20]. The mutation is responsive of the retention of intron 18 leading to a frameshift with a stop codon in position 2514.

Polymorphisms are presented in Table 2. We identified 92 polymorphisms in *PKD1 *and 6 in *PKD2 *. Two intronic variants in *PKD1 *were predicted to alter splicing but were considered as polymorphisms, c.2985+4G>A was present in 4 patients, a definite pathogenic mutation was identified in three and c.2985+5G>A was present in three individuals who already had a pathogenic mutation. The p.Glu107Asp novel substitution in *PKD2 *was classified polymorphism because it was found in 15 patients of the cohort, which is not consistent with a pathogenic mutation.

**Table 2:** Polymorphisms

DNA change	Protein change	Intron or exon	Fragment	Mutation type	Reference	Nb
***PKD1 ***						

c.107C>A	p.Pro36His	Exon 1	1	SUBSTITUTION	[37]	2
c.114C>T	p.Leu38Leu	Exon 1	1	SYNONYMOUS	Novel	1
c.1023C>T	p.Ala341Ala	Exon 5	5C	SYNONYMOUS	[9]	2
c.1119T>C	p.Leu373Leu	Exon 5	5C	SYNONYMOUS	[24]	1
c.1323G>A	p.Gly441Gly	Exon 6	6	SYNONYMOUS	[25]	1
c.1607-27C>T	Silent	Intron 7	8	INTRONIC	[10]	3
c.1714C>T	p.Pro572Ser	Exon 8	8	SUBSTITUTION	[10]	1
c.1850-4A>G	Silent	Intron 9	10	INTRONIC	[10]	2
c.2214C>G	p.Pro738Pro	Exon 11	11A	SYNONYMOUS	[18]	1
c.2216A>G	p.Gln739Arg	Exon 11	11A	SUBSTITUTION	[31]	5
c.2234C>G	p.Ala745Ala	Exon 11	11A	SYNONYMOUS	Novel	1
c.2700G>A	p.Pro900Pro	Exon 11	11B	SYNONYMOUS	[31]	1
c.2730C>T	p.Asp910Asp	Exon 11	11B	SYNONYMOUS	[31]	1
c.2854-5C>T	Silent	Intron 11	12	INTRONIC	[10]	1
c.2985+4G>A	Silent	Intron 12	12	INTRONIC	Novel	4
c.2985+5G>A	Silent	Intron 12	12	INTRONIC	Novel	3
c.2986-15C>T	Silent	Intron 12	13	INTRONIC	[38]	4
c.3063T>C	p.Gly1021Gly	Exon 13	13	SYNONYMOUS	[31]	8
c.3111A>G	p.Leu1037Leu	Exon 13	13	SYNONYMOUS	[9]	7
c.3275T>C	p.Met1092Thr	Exon 14	14	SUBSTITUTION	[10]	5
c.2395+53G>T	Silent	Intron 14	14	INTRONIC	Novel	1
c.3372C>T	p.Ala1124Ala	Exon 15	15A	SYNONYMOUS	[9]	8
c.3375C>T	p.Ser1125Ser	Exon 15	15A	SYNONYMOUS	[9]	8
c.3502C>T	p.Pro1168Ser	Exon 15	15A	SUBSTITUTION	[10]	2
c.4018C>T	p.Arg1340Trp	Exon 15	15B	SUBSTITUTION	[10]	1
c.4071G>T	p.Leu1357Leu	Exon 15	15C	SYNONYMOUS	[18]	1
c.4195T>C	p.Trp1399Arg	Exon 15	15C	SUBSTITUTION	[31]	8
c.4546G>A	p.Ala1516Thr	Exon 15	15D	SUBSTITUTION	[18]	1
c.4665A>C	p.Ala1555Ala	Exon 15	15D	SYNONYMOUS	[31]	11
c.4674G>A	p.Thr1558Thr	Exon 15	15D	SYNONYMOUS	[38]	4
c.5051C>T	p.Ser1684Leu	Exon 15	15E	SUBSTITUTION	[10]	1
c.5172C>T	p.Ala1724Ala	Exon 15	15E	SYNONYMOUS	[39]	11
c.5763G>A	p.Leu1921Leu	Exon 15	15G	SYNONYMOUS	[9]	1
c.6927C>T	p.Gly2309Gly	Exon 16	16	SYNONYMOUS	[39]	2
c.7165T>C	p.Leu2389Leu	Exon 17	17	SYNONYMOUS	[31]	7
c.7209+16G>A	Silent	Intron 17	17	INTRONIC	Novel	13
c.7210C>T	p.Arg2404Trp	Exon 18	18	SUBSTITUTION	CRISP unpublised	1
c.7441C>T	p.Leu2481Leu	Exon 18	18	SYNONYMOUS	[31]	11
c.7642G>C	p.Glu2548Gln	Exon 19	19	SUBSTITUTION	[39]	1
c.7703+24C>A	Silent	Intron 19	19	INTRONIC	[18]	1
c.7708T>C	p.Leu2570Leu	Exon 20	20	SYNONYMOUS	[32]	8
c.7863+47T>G	Silent	Intron 20	20	INTRONIC	[10]	9
c.7913A>G	p.His2638Arg	Exon 21	21	SUBSTITUTION	[38]	8
c.8016+13delG	Silent	Intron 21	21	INTRONIC	Novel	1
c.8016+26T>C	Silent	Intron 21	21	INTRONIC	[30]	8
c.8016+71G>A	Silent	Intron 21	21	INTRONIC	Novel	1
c.8016+77C>T	Silent	Intron 21	21	INTRONIC	Novel	1
c.8020C>T	p.Pro2674Ser	Exon 22	22	SUBSTITUTION	[40]	2
c.8087T>G	p.Leu2696Arg	Exon 22	22	SUBSTITUTION	[41]	12
c.8123C>T	p.Thr2708Met	Exon 22	22	SUBSTITUTION	[40]	1
c.8161+21T>C	Silent	Intron 22	22	INTRONIC	[30]	13
c.8161+23C>T	Silent	Intron 22	22	INTRONIC	Novel	9
c.8161+24C>G	Silent	Intron 22	22	INTRONIC	Novel	11
c.8161+25A>G	Silent	Intron 22	22	INTRONIC	Novel	11
c.8161+29del18	Silent	Intron 22	22	INTRONIC	Novel	1
c.8161+30C>G	Silent	Intron 22	22	INTRONIC	Novel	12
c.8161+31C>T	Silent	Intron 22	22	INTRONIC	Novel	12
c.8161+38G>A	Silent	Intron 22	22	INTRONIC	[30]	10
c.8161+39T>C	Silent	Intron 22	22	INTRONIC	[10]	1
c.8161+41C>T	Silent	Intron 22	22	INTRONIC	Novel	1
c.8161+42C>G	Silent	Intron 22	22	INTRONIC	Novel	10
c.8161+46ins18	Silent	Intron 22	22	INTRONIC	Novel	1
c.8644T>A	p.Trp2882Arg	Exon 23	23B	SUBSTITUTION	[18]	1
c.8681_8689del9	p.Ala2894_Ser2896del	Exon 23	23B	DELETION	[11]	1
c.8898G>C	p.Glu2966Asp	Exon 24	24	SUBSTITUTION	[42]	1
c.8913T>C	p.Ala2971Ala	Exon 24	24	SYNONYMOUS	[42]	2
c.8948+17A>G	Silent	Intron 24	24	INTRONIC	[18]	1
c.9195G>C	p.Val3065Val	Exon 25	25	SYNONYMOUS	Novel	1
c.9196T>C	p.Phe3066Leu	Exon 25	25	SUBSTITUTION	[43]	1
c.9260C>G	p.Thr3087Thr	Exon 26	26	SYNONYMOUS	Novel	1
c.9270C>T	p.Val3090Val	Exon 26	26	SYNONYMOUS	[39]	2
c.9330T>C	p.Pro3110Pro	Exon 26	26	SYNONYMOUS	[44]	12
c.9569-13T>C	Silent	Intron 27	28	INTRONIC	[10]	5
c.9712+30T>G	Silent	Intron 28	28	INTRONIC	Novel	2
c.10050+54A>G	Silent	Exon 30	30	INTRONIC	[10]	5
c.10170+14T>C	Silent	Intron 31	31	INTRONIC	[45]	1
c.10225G>C	p.Val3409Leu	Exon 33	33	SUBSTITUTION	[18]	1
c.10368C>T	p.Ala3456Ala	Exon 33	33	SUBSTITUTION	[18]	1
c.10406-4C>T	Silent	Intron 33	34	INTRONIC	Novel	1
c.10535C>T	p.Ala3512Val	Exon 35	35	SUBSTITUTION	[44]	4
c.10768C>T	p.Leu3590Leu	Exon 36	36	SYNONYMOUS	[46]	5
c.11376G>C	p.Ser3792Ser	Exon 40	40	SYNONYMOUS	[40]	1
c.11537+5_+6insGGG	Silent	Intron 41	41	INTRONIC	[39]	1
c.11682C>T	p.Ser3894Ser	Exon 42	42	SYNONYMOUS	[10]	2
c.11916C>T	p.Arg3972Arg	Exon 43	43	SYNONYMOUS	[39]	5
c.12138+22delG	Silent	Intron 44	44	INTRONIC	[47]	1
c.12176C>T	p.Ala4059Val	Exon 45	45	SUBSTITUTION	[48]	3
c.12201T>C	p.Pro4067Pro	Exon 45	45	SYNONYMOUS	Novel	9
c.12276A>G	p.Ala4092Ala	Exon 45	45	SYNONYMOUS	[31]	5
c.12409C>T	p.Leu4137Leu	Exon 45	45	SYNONYMOUS	[49]	1
c.12630T>C	p.Pro4210Pro	Exon 46	46A	SYNONYMOUS	[46]	1
c.12765C>T	p.Pro4255Pro	Exon 46	46B	SYNONYMOUS	[50]	1

***PKD2 ***						

c.83G>C	p.Arg28Pro	Exon 1	1A	SUBSTITUTION	[51]	1
c.321A>T	p.Glu107Asp	Exon 1	1B	SUBSTITUTION	Novel	15
c.420G>A	p.Gly140Gly	Exon 1	1B	SYNONYMOUS	[52]	6
c.568G>A	p.Ala190Thr	Exon 1	1B	SUBSTITUTION	[25]	3
c.844-22G>A	Silent	Intron 3	4	INTRONIC	[51]	1
c.1445T>G	p.Phe482Cys	Exon 6	6	SUBSTITUTION	[53]	1

Nb: number of patients carrying the sequence variant.

We found 12 indeterminate sequence variants in *PKD1 *and none in *PKD2 *. They are presented in Table 3. The majority of these variants were substitutions; their scoring is presented in Additional file 2. The small in frame insertion p.Asp3781_Val3782insGlu was considered as indeterminate because the patient had another indeterminate variant (p.Arg4276Trp) and we could not be sure of its pathogenicity. The three other indeterminate sequence variants (p.Ala2704Val, p.Ser2935Phe p.His3559Pro) are the sole mutation with potential pathogenicity observed in the three individuals screened.

**Table 3:** Indeterminate variants

DNA change	Protein change	Intron or exon	Fragment	Mutation type	Reference	Nb
***PKD1 ***						

c.854C>T	p.Ala285Val	Exon 5	5B	SUBSTITUTION	Novel	**1**
c.4439A>G	p.Glu1480Gly	Exon 15	15C	SUBSTITUTION	Novel	**1**
c.5848G>A	p.Val1950Met	Exon 15	15G	SUBSTITUTION	Novel	**1**
c.8110C>T	p.Ala2704Val	Exon 22	22	SUBSTITUTION	Novel	**1**
c.8803C>T	p.Ser2935Phe	Exon 24	24	SUBSTITUTION	Novel	**1**
c.9730G>A	p.Arg3244His	Exon 29	29	SUBSTITUTION	Novel	**1**
c.10043G>A	p.Arg3348Gln	Exon 30	30	SUBSTITUTION	Novel	**1**
c.10876A>C	p.His3559Pro	Exon 36	36	SUBSTITUTION	Novel	**2**
c.11108G>C	p.Ser3703Thr	Exon 38	38	SUBSTITUTION	Novel	**4**
c.11344_11345insAAG	p.Asp3781_Val 3782insGlu	Exon 40	40	INSERTION	Novel	**1**
c.11698C>T	p.Leu3897Phe	Exon 42	42	SUBSTITUTION	Novel	**1**
c.12826C>T	p.Arg4276Trp	Exon 46	46B	SUBSTITUTION	[54]	**1**

Nb: number of patients carrying the sequence variant.

## Discussion

We describe the first sequence variants pre-screening method for *PKD1 *and *PKD2 *using HRM analysis. This is the first use of this technique in genetic nephrology. This method has a good diagnosis rate as evidenced by the identification of a mutation in 75% of our cohort (including definitely pathogenic and probably pathogenic mutations), which is the mean diagnosis rate reported with direct sequencing of the two genes [24]. In comparison, Rossetti et al found a diagnosis rate of 62.9% with direct sequencing [11] and 67% with dHPLC [10]. In addition a recent report of Hoefele et al found a mutation detection efficiency of 64,5% by direct sequencing [25].

More than 81% of amplicons suspected to have a sequence variant after HRM analysis carried one after sequencing, confirming the specificity of this technology. Overall, less than 15% of the fragments of *PKD1 *and 1.5% of the *PKD2 *fragments analyzed by HRM were sequenced. Moreover, we screened in a blind procedure four patients with previously known mutations [20] and identified all of them. Thus, HRM is efficient, sensitive and specific for mutation screening in ADPKD genes. HRM analysis reduced drastically the need for systematic sequencing and the time of sequence analysis.

We describe 52 new sequence variants in *PKD1 *: 18 were classified as mutation, no mutation was detected in more than one individual, 23 as polymorphism and 11 as indeterminate variant. We report one new mutation and one polymorphism in *PKD2 *.

Large deletions or duplications represent 4% of ADPKD pathogenic variants [26]. HRM technology allows genomic qPCR screening to be performed for large deletions/duplications in the same run. Successful simultaneous analysis by HRM and genomic qPCR has been described for *MLH1 *gene [27]. Therefore, it is likely that HRM and genomic qPCR can be performed simultaneously for *PKD1 *and *PKD2 *, which will require the introduction of control DNA and a reference gene in the microtiter plate. This will improve molecular diagnosis by directly identifying large deletions/duplications that have not been identified by HRM analysis or with sequencing. Therefore, HRM analysis could prove to be a highly integrated molecular diagnosis tool.

HRM analysis is a high throughput technique for sequence variants screening *PKD1 *and *PKD2 *. The format of PCR analysis in 96 or 394 well plates allows the process to be automated after DNA extraction and the first round of LR PCR for *PKD1 *. A major advantage of HRM is a drastic diminution in the needs for sequencing to identify a sequence variation. HRM could be more cost effective than direct sequencing for identification of molecular anomalies in ADPKD. Comparing to dHPLC, HRM is a closed tube and a non destructive technique. The PCR and analytical steps are in the same run with HRM. During dHPLC several separate steps require PCR products manipulation and can induce mistakes. After analysis, in HRM the PCR product can be directly sequenced; in dHPLC the PCR product is destroyed during the run and a new PCR must be done for sequencing. In our facility, the price for one amplicon analysis with HRM is 1.52 $ (1.10 €) compared to 4.64 $ (3.36 €) with dHPLC [28]. In addition, HRM allows large fragment rearrangements detection in the same step. This technique is very competitive compared to dHPLC. HRM is time sparing, provides greater security (closed tube during the whole process) and is cheaper. Assuming bidirectional sequencing costs 13.8 $ (10 €) per fragment, the cost for mutation screening in *PKD1 *and *PKD2 *with direct sequencing costs 966 $ (700 €) (70 fragments). With HRM, 70 fragments × 1.52 $ plus sequencing of positive fragments (i.e. 9 fragments × 13.8 $), the total cost is of 230.46 $ (167 €). Furthermore, we do not consider here the technician time sparing provided by HRM in this cost analysis. Thus, acquiring the needed hardware for HRM analysis is easily paid off. HRM pre-screening is cost and time efficient facing direct sequencing in ADPKD genes.

The technique efficiency will soon be further improved with the search of conditions allowing the screening of exons 1 of *PKD1 *and *PKD2 *and exons 42 and 43 of *PKD1 *. We were not able to conduct HRM analysis of these exons because the denaturation curves could not be analyzed. We suspected this failure is related to the high GC contents of the DNA sequence of exon 1 of *PKD1 *and *PKD2 *. For exon 42 and 43 of *PKD1 *, we suspected the formation of secondary structures not solved by denaturation temperature. HRM is a rather young technology and we can expect important developments increasing the efficiency of this sensitive and specific method of screening [28].

Molecular diagnosis of ADPKD is important for improving the care of patients [29]. Renal transplantation from a related living donor at risk of ADPKD is a concern since clinical and ultrasonographic diagnosis criteria fail to identify donors with ADPKD when they are aged less than 40 years [30]. Molecular diagnosis could be of use in this area as well as identifying mutations in ambiguous phenotypes [31], for example when the family history is unknown. Genetic diagnosis also results in a more accurate prognosis [32] as the genes mutated in ADPKD are the prognostic factors linked to kidney survival [33]. In addition, therapies development will probably require an accurate diagnosis in young adults to initiate treatment before significant renal changes have occurred. HRM could be used as a quick and cost effective genetic test for individuals included in clinical trials.

## Conclusions

HRM analysis is an efficient, sensitive, specific and cost effective strategy to identify mutations in *PKD1 *and *PKD2 *.

## Competing interests

All authors declared to have non-financial competing interests in relation to this manuscript.

## Authors' contributions

SB carried out the molecular genetic studies, participated in the sequence alignment and drafted the manuscript. YB participated to recruitment of patients. MF participated in the design of the study and drafted the manuscript. SB conceived of the study, and participated in its design and coordination, and participated to recruitment of patients and helped to draft the manuscript. All authors read and approved the final manuscript.

## Pre-publication history

The pre-publication history for this paper can be accessed here:

http://www.biomedcentral.com/1471-2369/12/57/prepub

## Supplementary Material

Additional file 1 **Primers used for *PKD1 *and *PKD2 *analysis**. The file contains the sequence and characteristic of the primers used for amplification of *PKD1 *and *PKD2 *. Click here for file

Additional file 2 **Scoring details of novel coding sequence variations**. The file contains the score to evaluate the pathogenicity of the new sequence variations described in the text. Click here for file
