# Supplementary material for: High Resolution Melt analysis for mutation screening in PKD1 and PKD2
Source: BMC Nephrol. 2011 Oct 18;12:57. doi: 10.1186/1471-2369-12-57 (PMC3206831; doi:10.1186/1471-2369-12-57)
Supplement: Additional file 1 — Primers used for PKD1 and PKD2 analysis. The file contains the sequence and characteristic of the primers used for amplification of PKD1 and PKD2 . [file 1471-2369-12-57-S1.DOC]

**Additional file 1: Primers used for *PKD1* and *PKD2* analysis. (**F: forward; R: reverse)

**Primers used for PKD1 long range PCR**

| **Primer** | **Sequence** | **Nb of nucléotides** | **Denaturation temperature** | **Amplicon** | **Exon/LR** | **Ref.** |
| --- | --- | --- | --- | --- | --- | --- |
| PKD1-LR1-F | CGCAGCCTTACCATCCACCT | 20 |  | 2278bp | 1 | Tan 2008 |
| PKD1-LR1-R | TCATCGCCCCTTCCTAAGCA | 20 |  |
| PKD1-LR2-F | CCAGCTCTCTGTCTACTCACCTCCGCATC | 29 |  | 8725bp | 2-12 | Tan 2008 |
| PKD1-LR2-R | CCACGGTTACGTTGTAGTTCACGGTGACG | 29 |  |
| PKD1-LR3-F | TGGAGGGAGGGACGCCAATC | 20 |  | 4386bp | 13-15G | Tan 2008 |
| PKD1-LR3-R | GTCAACGTGGGCCTCCAAGT | 20 |  |
| PKD1-LR4-F | ATCCCTGGGGGTCCTACCATCTCTTA | 26 |  | 5253bp | 15C-21 | Tan 2008 |
| PKD1-LR4-R | ACACAGGACAGAACGGCTGAGGCTA | 25 |  |
| PKD1-LR5-F | ATGCTTAGTGAGGAGGCTGTGGGGGTCCA | 29 |  | 7583bp | 22-32 | Tan 2008 |
| PKD1-LR5-R | TCGGCAAGGACCTGCTGGATCAGGTCTTC | 29 |  |
| PKD1-LR6-F | ACCTTCCCTCTAGGGAGGGAGCA | 23 |  | 7838bp | 36-46 | None |
| PKD1-LR6-R | GGCCAAGCTCGCATCCAAGCA | 21 |  |

**Primers used for *PKD1* analysis:**

| **Fragment** | Primer sequence | | **Amplicon length** | **Corresponding long range fragment** | **Denaturation temperature** | **Annealing temperature** | **HRM initial temperature** | **HRM final temperature** | **Reference** |
| --- | --- | --- | --- | --- | --- | --- | --- | --- | --- |
| Exon 1 | F | GCGTCGCTCAGCAGCAGGT | 448 bp | LR1 | - | - | - | - | Tan 2009 |
|  | R | GCCCGCGTCCTGCTTCCC |  |  |  |  |  |  |  |
| Exon 2 | F | GGGATGCTGGCAATGTGTGGGAT | 243 bp | LR2 | 96°C | 70°C | 96°C | 98°C | Tan 2009 |
|  | R | CCCACAGCTGAGCAGCAAGAG |  |  |  |  |  |  |  |
| Exon 3 | F | GCACCGCCTCTTGCTGCTCA | 216 bp | LR2 | 96°C | 65°C | 96°C | 98°C | Tan 2009 |
|  | R | GGACCAACTGGGAGGGCAGAA |  |  |  |  |  |  |  |
| Exon 4 | F | GGCGGTGCTGTCAGGGTG | 367 bp | LR2 | 96°C | 70°C | 96°C | 98°C | Tan 2009 |
|  | R | CCAGAGAGGCCTTCCTGAGC |  |  |  |  |  |  |  |
| Exon 5A | F | TGGAGCCAGGAGGAGCAGAA | 262 bp | LR2 | 96°C | 70°C | 96°C | 98°C | Rossetti 2002 |
|  | R | CAGAGGGACAGGCAGGCAAA |  |  |  |  |  |  |  |
| Exon 5B | F | AGCCCTCCAGTGCCTCCTTT | 303 bp | LR2 | 96°C | 67°C | 96°C | 98°C | Rossetti 2002 |
|  | R | GCACGGCCGTCACGTGATAG |  |  |  |  |  |  |  |
| Exon 5C | F | TGGGACTTCGGAGACGGCT | 310 bp | LR2 | 96°C | 70°C | 96°C | 98°C | Rossetti 2002 |
|  | R | GAGTGGGCAGCAGACACTCA |  |  |  |  |  |  |  |
| Exon 6 | F | GTGTCTGCTGCCCACTCCC | 391 bp | LR2 | 96°C | 70°C | 96°C | 98°C | Tan 2009 |
|  | R | CTCCTTCCTCCTGAGACTCCC |  |  |  |  |  |  |  |
| Exon 7 | F | GCTGCTGTGAGGGTGGGAGGA | 358 bp | LR2 | 96°C | 70°C | 96°C | 98°C | Tan 2009 |
|  | R | TCCACCGCGGGCGCTCGGCA |  |  |  |  |  |  |  |
| Exon 8 | F | CTGGGCTGAGGAGGAGGG | 348 bp | LR2 | 96°C | 70°C | 96°C | 98°C | Tan 2009 |
|  | R | GGGCACAAGCAACATTAAGGCCC |  |  |  |  |  |  |  |
| Exon 9 | F | CCTCTTCCTGGGAAGTTCGGGT | 312 bp | LR2 | 96°C | 67°C | 96°C | 98°C | Tan 2009 |
|  | R | ACTCTGGTGGCCACAGGACCA |  |  |  |  |  |  |  |
| Exon 10 | F | GCAGGCAGTTGGGCATCTCTG | 389 bp | LR2 | 96°C | 70°C | 96°C | 98°C | Tan 2009 |
|  | R | GACCCTGGGCAGCAGACAG |  |  |  |  |  |  |  |
| Exon 11A | F | GTGTGGCTGACGAAGCGGG | 581 bp | LR2 | 96°C | 70°C | 96°C | 98°C | Tan 2009 |
|  | R | CCGTGGCGTTGGCACCAG |  |  |  |  |  |  |  |
| Exon 11B | F | CGCTATGAGGTCCGGGCAG | 565 bp | LR2 | 96°C | 70°C | 96°C | 98°C | Tan 2009 |
|  | R | CCCTCACTGGGAAGCCAGG |  |  |  |  |  |  |  |
| Exon 12 | F | TGTGTCCAGGAGGCGACAG | 274 bp | LR2 | 96°C | 67°C | 96°C | 98°C | Tan 2009 |
|  | R | AGAGGTGAAGGTGGAGCCCG |  |  |  |  |  |  |  |
| Exon 13 | F | CTGCCACCTGGGCTCACTG | 244 bp | LR3 | 96°C | 70°C | 98°C | 99°C | Rossetti 2002 |
|  | R | TGCCCACCCCAAACCGGC |  |  |  |  |  |  |  |
| Exon 14 | F | CTGCAGAGTGGAGCCTCGG | 342 bp | LR3 | 96°C | 65°C | 98°C | 99°C | Tan 2009 |
|  | R | GTCACAGTGAGGGCTGTTGGG |  |  |  |  |  |  |  |
| Exon 15A | F | AGGTGCAGCCGTTCACCC | 547 bp | LR3 | 96°C | 65°C | 98°C | 99°C | Tan 2009 |
|  | R | TCGAAGGTCCACGTGATGT |  |  |  |  |  |  |  |
| Exon 15B | F | GACATGAGCCTGGCCGTGG | 490 bp | LR3 | 96°C | 70°C | 98°C | 99°C | Tan 2009 |
|  | R | CCACCTCTGGCTCCACGCA |  |  |  |  |  |  |  |
| Exon 15C | F | CACGCGGAGCGGCACGTT | 496 bp | LR3 | 96°C | 70°C | 98°C | 99°C | Tan 2009 |
|  | R | GGTGACCTCCGGACCCTC |  |  |  |  |  |  |  |
| Exon 15D | F | TCTGCTGTGGGCCGTGGG | 492 bp | LR3 | 96°C | 70°C | 98°C | 99°C | Tan 2009 |
|  | R | CTGTACCGTGTGGTTGGTGGG |  |  |  |  |  |  |  |
| Exon 15E | F | ACAGCATCTTCGTCTATGTCCTG | 492 bp | LR4 | 98°C | 65°C | 98°C | 99°C | Tan 2009 |
|  | R | GGTTCCCTGCCGTCATGGTG |  |  |  |  |  |  |  |
| Exon 15F | F | GGGCTGAGCTGGGAGACCT | 483 bp | LR4 | 98°C | 65°C | 98°C | 99°C | Tan 2009 |
|  | R | GACAGCTGAGCCGGCAGC |  |  |  |  |  |  |  |
| Exon 15G | F | CTGTGGGCCAGCAGCAAGGT | 483 bp | LR4 | 98°C | 65°C | 98°C | 99°C | Tan 2009 |
|  | R | CGTGCGGTTCTCACTGCCCA |  |  |  |  |  |  |  |
| Exon 15H | F | GACGTCACCTACACGCCCG | 493 bp | LR4 | 98°C | 65°C | 98°C | 99°C | Tan 2009 |
|  | R | CCTCCCAGCGGTACTCAGTCT |  |  |  |  |  |  |  |
| Exon 15I | F | GATGCGGCGATCACAGCGCA | 525 bp | LR4 | 98°C | 65°C | 98°C | 99°C | Tan 2009 |
|  | R | GGCCAGCCCTGGTGGCAA |  |  |  |  |  |  |  |
| Exon 16 | F | GGCCCGTCCTCAGTGCCT | 378 bp | LR4 | 98°C | 65°C | 98°C | 99°C | Tan 2009 |
|  | R | GCGGCCTCCACCAGCACTA |  |  |  |  |  |  |  |
| Exon 17 | F | GAGGTAACCCCACTCCCACG | 203 bp | LR4 | 98°C | 65°C | 98°C | 99°C | Rossetti 2002 |
|  | R | ATCCCCAGCCCGCCCACAC |  |  |  |  |  |  |  |
| Exon 18 | F | AGAGGGTTGCGCCCCCTC | 353 bp | LR4 | 98°C | 65°C | 98°C | 99°C | Rossetti 2002 |
|  | R | ATCCCGCTGCTCCCCCCACGCAGG |  |  |  |  |  |  |  |
| Exon 19 | F | GCACGGGTGAGTGCAGGC | 387 bp | LR4 | 98°C | 70°C | 98°C | 99°C | Tan 2009 |
|  | R | CCAAAGACCTACGAGCAGAGGG |  |  |  |  |  |  |  |
| Exon 20 | F | TGGGAGCCGCTGTGGTCG | 352 bp | LR4 | 98°C | 65°C | 98°C | 99°C | Tan 2009 |
|  | R | CCGGGATGAGCCCTCTGCAA |  |  |  |  |  |  |  |
| Exon 21 | F | CGTCTAGCACGTAACTGCACCC | 353 bp | LR4 | 98°C | 65°C | 98°C | 99°C | Tan 2009 |
|  | R | GGCTCAGCTCCTCGGCCA |  |  |  |  |  |  |  |
| Exon 22 | F | CAGGTGAGGACCCGTGTAGAGA | 295 bp | LR5 | 96°C | 70°C | 96°C | 97°C | Tan 2009 |
|  | R | GGGAGGAGGGAGGCAGAG |  |  |  |  |  |  |  |
| Exon 23A | F | CCCTCCCTCTACCTCCCTGTC | 399 bp | LR5 | 96°C | 70°C | 96°C | 97°C | Tan 2009 |
|  | R | GCCAAAGGGAAAGGGATTGGAGT |  |  |  |  |  |  |  |
| Exon 23B | F | GGCTGCCACTTCTCCATCCC | 469 bp | LR5 | 96°C | 70°C | 96°C | 97°C | Tan 2009 |
|  | R | GACACCCATGGAAGCCCTACG |  |  |  |  |  |  |  |
| Exon 24 | F | CGTGGCAGAGGGTGGGCT | 344 bp | LR5 | 96°C | 70°C | 96°C | 97°C | Tan 2009 |
|  | R | CTCGCTGCCTGCCGTCCC |  |  |  |  |  |  |  |
| Exon 25 | F | GGCTCTGAGACTGCGACATCCAA | 407 bp | LR5 | 96°C | 70°C | 96°C | 97°C | Tan 2009 |
|  | R | CAAGCTGTGCCTTCTCAGGATAG |  |  |  |  |  |  |  |
| Exon 26 | F | CTGCAGAGTCGAGGAGGGC | 390 bp | LR5 | 96°C | 70°C | 96°C | 97°C | Tan 2009 |
|  | R | CTTGTTCTGACGCCTGCGACG |  |  |  |  |  |  |  |
| Exon 27 | F | GCTGAGATGACTTGCCTGGGATG | 316 bp | LR5 | 96°C | 70°C | 96°C | 97°C | Tan 2009 |
|  | R | GAGGTCAGGCTCGCAGGG |  |  |  |  |  |  |  |
| Exon 28 | F | CCCTGCCAAGCTCTGCCC | 292 bp | LR5 | 96°C | 70°C | 96°C | 97°C | Tan 2009 |
|  | R | ACTGCAGGAGGCCACGGG |  |  |  |  |  |  |  |
| Exon 29 | F | CTCCGTGGGAGGTTGGGCA | 366 bp | LR5 | 96°C | 70°C | 96°C | 97°C | Tan 2009 |
|  | R | TGGAGGAGAGGAGGCCACACA |  |  |  |  |  |  |  |
| Exon 30 | F | CTACAGGTGGGTGCCGTAGG | 326 bp | LR5 | 96°C | 70°C | 96°C | 97°C | Tan 2009 |
|  | R | CGCCTTTCCCTCTGGCTGC |  |  |  |  |  |  |  |
| Exon 31 | F | CGGGCTCTGTCCTGTCTGC | 308 bp | LR5 | 96°C | 70°C | 96°C | 97°C | Tan 2009 |
|  | R | CAAAGGCCTGCTGAGAGGTGC |  |  |  |  |  |  |  |
| Exon 32 | F | GGTCCTGGGCTGGGCTGG | 210 bp | LR5 | 95°C | 72°C | 96°C | 97°C | Tan 2009 |
|  | R | CCCAGCAAGGACACGCAGC |  |  |  |  |  |  |  |
| Exon 33 | F | GGGTGGGCTGTGTGTGTGAC | 286 bp | None | 95°C | 64°C | 96°C | 98°C | Rossetti 2002 |
|  | R | GCAAGGGTGAGCTTCAGAGCC |  |  |  |  |  |  |  |
| Exon 34 | F | GCCCACCCTATGCCTCCTG | 154 bp | None | 95°C | 64°C | 96°C | 98°C | Rossetti 2002 |
|  | R | AATCCCCCCTCCCCCGAGAGCCGG |  |  |  |  |  |  |  |
| Exon 35 | F | CAAGAGGCTCAAGAAACTGCCCG | 313 bp | None | 95°C | 64°C | 96°C | 98°C | Tan 2009 |
|  | R | GCAGACCCTCCACCAGTCCT |  |  |  |  |  |  |  |
| Exon 36 | F | CCTCCCTGTGAGCTGCCTCTC | 280 bp | LR6 | 95°C | 64°C | 96°C | 98°C | Rossetti 2002 |
|  | R | GGCCTGTAGCCTACCCCTGG |  |  |  |  |  |  |  |
| Exon 37 | F | TCCATCACGGGGGACCCCTCT | 325 bp | LR6 | 95°C | 64°C | 96°C | 98°C | Rossetti 2002 |
|  | R | AAAGGGGGACAGGAGTGTCCT |  |  |  |  |  |  |  |
| Exon 38 | F | GCCGTTGCCAAAGCCCTGCT | 281 bp | LR6 | 95°C | 64°C | 96°C | 97°C | Tan 2009 |
|  | R | CCCTAGGGTCTGGCTGGACTA |  |  |  |  |  |  |  |
| Exon 39 | F | GCCAGCAGGGCAGTGGGA | 314 bp | LR6 | 95°C | 64°C | 96°C | 97°C | Tan 2009 |
|  | R | CAGCTAGGGAGCAGGGCTGA |  |  |  |  |  |  |  |
| Exon 40 | F | GTGGCGCCGAACCAGAGC | 350 bp | LR6 | 95°C | 64°C | 96°C | 97°C | Tan 2009 |
|  | R | CCAGCCCTTCCGGCACCC |  |  |  |  |  |  |  |
| Exon 41 | F | CGGCCTCCTGACCAGCCTGGCTC | 330 bp | LR6 | 95°C | 64°C | 96°C | 97°C | Rossetti 2002 |
|  | R | TAGGCCAGCGGGGGCCGGAGGAGTG |  |  |  |  |  |  |  |
| Exon 42 | F | CCTCAGCCACGCCTGCACT | 341bp | LR6 | - | - | - | - | Tan 2009 |
|  | R | GGGTGAGACGCTGCCGGG |  |  |  |  |  |  |  |
| Exon 43 | F | CCCGGCAGCGTCTCACCC | 550 bp | LR6 | - | - | - | - | Tan 2009 |
|  | R | CAGCCTGCGGACGAGAAATCTG |  |  |  |  |  |  |  |
| Exon 44 | F | TGGCCTGGCGGCCTCGCT | 347 bp | LR6 | 95°C | 64°C | 96°C | 97°C | Tan 2009 |
|  | R | CAGGAAGACACGAGCTGCGG |  |  |  |  |  |  |  |
| Exon 45 | F | AGCTCAGCTGTACGCCCTCA | 379 bp | LR6 | 95°C | 64°C | 96°C | 97°C | Rossetti 2002 |
|  | R | TGTCCCTCTCCCCCCCACTG |  |  |  |  |  |  |  |
| Exon 46A | F | GGAGAGGGACACGCCCTG | 332 bp | LR6 | 95°C | 62°C | 96°C | 97°C | Tan 2009 |
|  | R | ACGTCCTCTGTGGCCTGGTTG |  |  |  |  |  |  |  |
| Exon 46B | F | TGAGCCCTCCCGCCTCCAA | 376 bp | LR6 | 97°C | 66°C | 98°C | 99°C | Tan 2009 |
|  | R | ATTCTGCCTGGCCCTCGGCCTT |  |  |  |  |  |  |  |

**Primers used for *PKD2* analysis:**

| **Fragment** | **Primer sequence** | | **Amplicon length** | **Denaturation temperature** | **Annealing temperature** | **HRM initial temperature** | **HRM final temperature** | **Reference.** |
| --- | --- | --- | --- | --- | --- | --- | --- | --- |
| Exon 1A | F | CCAGTGACCGCGATGGTG | 367 bp | - | - | - | - | Tan 2009 |
|  | R | GCCACTCTACGTCCATCTCC |  |  |  |  |  |  |
| Exon 1B | F | GTGGAGCCGCGATAACCC | 385 bp | - | - | - | - | Tan 2009 |
|  | R | GTGGAGCCGCGATAACCC |  |  |  |  |  |  |
| Exon 2 | F | TTTGCCATTCATGAGATTTC | 274 bp | 90°C | 60°C | 88°C | 92°C | Tan 2009 |
|  | R | CCCTCTGGTGCATACACAC |  |  |  |  |  |  |
| Exon 3 | F | GTGAAGGCTGCTGGTATG | 279 bp | 90°C | 60°C | 88°C | 92°C | Tan 2009 |
|  | R | ATGCATTGAAATGATGAAGC |  |  |  |  |  |  |
| Exon 4 | F | TTGGTTATGCAAACGATG | 392 bp | 90°C | 60°C | 88°C | 92°C | Tan 2009 |
|  | R | GGGAGTTCAGAGAATAAATG |  |  |  |  |  |  |
| Exon 5 | F | GCCTCAAGTGTTCCACTGAT | 361 bp | 90°C | 60°C | 88°C | 92°C | Hayashi 1997 |
|  | R | AGGTTTTTCTGGCTAACCCCAG |  |  |  |  |  |  |
| Exon 6 | F | GGACATCCATTCCTGGCTG | 379 bp | 90°C | 60°C | 88°C | 92°C | Tan 2009 |
|  | R | ACAATGCTGAGGAGATCAAAG |  |  |  |  |  |  |
| Exon 7 | F | GGTGAGCCCTTATAATTAATAC | 383 bp | 90°C | 60°C | 88°C | 92°C | Tan 2009 |
|  | R | GCTGGTCACTTGAATTTC |  |  |  |  |  |  |
| Exon 8 | F | TCATCCATGTTGTAACCTG | 401 bp | 90°C | 60°C | 88°C | 92°C | Tan 2009 |
|  | R | ATAATTGGTGGTCATATAGC |  |  |  |  |  |  |
| Exon 9 | F | AAATGTTGCATCAACTAGTG | 274 bp | 90°C | 60°C | 88°C | 92°C | Tan 2009 |
|  | R | TGATAGGACATAAAGTGTGAG |  |  |  |  |  |  |
| Exon 10 | F | TCATAAAGCACTCAGATTAGG | 290 bp | 90°C | 60°C | 88°C | 92°C | Tan 2009 |
|  | R | AAATCTGGGTGAAACAATG |  |  |  |  |  |  |
| Exon 11 | F | GTTACTACTGTGAATGGAAAG | 290 bp | 90°C | 60°C | 88°C | 92°C | Tan 2009 |
|  | R | CCCTTGGGCTAGAAATAC |  |  |  |  |  |  |
| Exon 12 | F | TGATGTCTCTGTGTTGAGG | 250 bp | 90°C | 60°C | 88°C | 92°C | Tan 2009 |
|  | R | TTGATACATCTGTGGTGTTG |  |  |  |  |  |  |
| Exon 13 | F | CCAGTTCCTGCTTGCCCAAGTC | 316 bp | 90°C | 60°C | 88°C | 92°C | Tan 2009 |
|  | R | GAGGGAACTGCCTGGTCTCATG |  |  |  |  |  |  |
| Exon 14 | F | AAAGACAATGACAAGCACTTTG | 278 bp | 90°C | 60°C | 88°C | 92°C | Tan 2009 |
|  | R | TCAAATACAACTGTCAGCAAC |  |  |  |  |  |  |
| Exon 15 | F | ATTTGGTCCCTGGACTTC | 400 bp | 90°C | 60°C | 88°C | 92°C | Tan 2009 |
|  | R | GTGGTCAGGGCATATAAATAG |  |  |  |  |  |  |
