# Supplementary material for: High Resolution Melt analysis for mutation screening in PKD1 and PKD2
Source: BMC Nephrol. 2011 Oct 18;12:57. doi: 10.1186/1471-2369-12-57 (PMC3206831; doi:10.1186/1471-2369-12-57)
Supplement: Additional file 2 — Scoring details of novel coding sequence variations. The file contains the score to evaluate the pathogenicity of the new sequence variations described in the text. [file 1471-2369-12-57-S2.DOC]

| **DNA change** | **Protein change** | **Fragment** | **FINAL MUTATION INTERPRETATION** | **POLYPHEN** | | **Mutation scoring according to Rossetti et al. (Rossetti 2007)** | | | | | | | | | |
| --- | --- | --- | --- | --- | --- | --- | --- | --- | --- | --- | --- | --- | --- | --- | --- |
|  |  |  |  | **Mutation**  **prediction** | **Score** | **Clinical**  **significance** | **Total**  **Score** | **GD** | **GV** | **GD/GV** | **Substitution**  **found in**  **orthologue** | **Conserved**  **Domain** | **Mutation**  **in same**  **residue** | **Change**  **In**  **splicing** | **Other mutation**  **in same**  **patient** |
| c.854C>T | p.Ala285Val | 5B | Indeterminate | Possibly damaging | 0.373 | Indeterminate | -3 | 64 | 111 | -2 | - | - | - | - | -1 |
| c.2073G>A | p.Ala692Thr | 10 | Probably pathogenic | Probably damaging | 0.960 | Likely pathogenic | 6 | 58 | 27 | +4 | - | - | - | - | +2 |
| c.4439A>G | p.Glu1480Gly | 15C | Indeterminate | Probably damaging | 0.995 | Indeterminate | 1 | 98 | 65 | +3 | - | - | - | - | -2 |
| c.5610C>G | p.Asn1870Lys | 15F | Probably pathogenic | Probably damaging | 0.995 | Highly likely pathogenic | 14 | 94 | 0 | +6 | - | +4 | +2 | - | +2 |
| c.5848G>A | p.Val1950Met | 15G | Indeterminate | Possibly damaging | 0.298 | Indeterminate | 4 | 21 | 32 | -2 | - | +4 | - | - | +2 |
| c.8110C>T | p.Ala2704Val | 22 | Indeterminate | Benign | 0.002 | Indeterminate | -3 | 64 | 107 | -2 | - | - | - | - | -1 |
| c.8803C>T | p.Ser2935Phe | 24 | Indeterminate | Possibly damaging | 0.442 | Indeterminate | -1 | 155 | 145 | 0 | - | - | - | - | -1 |
| c.9403C>T | p.Thr3135Met | 27 | Probably pathogenic | Probably damaging | 0.999 | Highly likely pathogenic | 12 | 81 | 0 | +6 | - | +4 | - | - | +2 |
| c.9730G>A | p.Arg3244His | 29 | Indeterminate | Probably damaging | 0.878 | Likely neutral | -9 | 29 | 110 | -4 | - | - | - | - | -5 |
| c.10043G>A | p.Arg3348Gln | 30 | Indeterminate | Probably damaging | 0.999 | Indeterminate | 0 | 43 | 0 | +5 | - | - | - | - | -5 |
| c.10876A>C | p.His3559Pro | 36 | Indeterminate | Probably damaging | 0.999 | Likely neutral | -7 | 77 | 99 | -2 | - | - | - | - | -5 |
| c.11108G>C | p.Ser3703Thr | 38 | Indeterminate | Probably damaging | 0.956 | Likely neutral | -11 | 58 | 110 | -6 | -4 | - | - | - | -1 |
| c.11249G>A | p.Arg3750Gln | 39 | Probably pathogenic | Probably damaging | 0.999 | Likely pathogenic | 8 | 43 | 0 | +5 | - | +4 | - | - | -1 |
| c.11689C>T | p.Leu3897Phe | 42 | Indeterminate | Possibly damaging | 0.373 | Likely neutral | -7 | 22 | 32 | -2 | - | - | - | - | -5 |

**Additional file 2: Scoring details of novel coding sequence variations.**

GD: Grantham distance; GV: Grantham variation; GD/GV: matrix score from comparison between GD and GV.

Comparison with homologues was made with mouse (*mus* *musculus*), rat (*rattus* *norvegicus*), chicken (*gallus* *gallus*), frog (*xenopus* *laevis*), and fish (*takifugu* *rubripes*).

The scoring method is described by Rossetti et al. (Rossetti 2007).
